# Intraoperative Cryotherapy as a Local Adjuvant After Bone Curettage in Orthopedic Oncology: A Review of Modern Literature

**DOI:** 10.3390/jcm14228007

**Published:** 2025-11-12

**Authors:** Antonio D’Arienzo, Edoardo Ipponi, Fabio Cosseddu, Francesco Rosario Campo, Paolo Domenico Parchi, Lorenzo Andreani

**Affiliations:** Department of Orthopedics and Trauma Surgery, University of Pisa, Via Paradisa 2, 56124 Pisa, Italy

**Keywords:** bone tumor, complication, cryoprobes, cryosurgery, liquid nitrogen, recurrence, fracture

## Abstract

**Background**: Curettage is a well-established treatment for benign bone tumors. Among the adjuvant treatments available to minimize the risk of local recurrence after curettage, cryotherapy is one of the most used and documented. Our study aims to summarize the results of curettage and intraoperative cryotherapy for the treatment of bone tumors in the modern literature. **Methods**: We systematically reviewed the existing literature, searching for cases treated with intraoperative cryotherapy after bone curettage in orthopedic oncology. Articles from the PubMed and MEDLINE databases, published between January 2000 and January 2025, were included. Our research was conducted in accordance with PRISMA guidelines. Case reports were excluded. For each study, we recorded the number of cases, their histological diagnosis, the curettage technique, and the cryotherapy administration strategy. Complications and recurrence rates were recorded, as well as post-operative functional performance. **Results**: Twenty-two studies met our inclusion criteria. A total of 1451 cases with benign and low-grade malignant bone tumors were recorded. After a mean follow-up of 55.7 months, the mean recurrence rate was 7.4% and the global complication rate was 8.7%. The mean MSTS score was 27.8. **Conclusions**: The combination of curettage and intra-operative cryotherapy, administered with either open or closed contact techniques, can be effective in eradicating benign and low-grade bone tumors and has low complication rates and a limited impact on patients’ functionality.

## 1. Introduction

Benign bone tumors represent a heterogeneous group of musculoskeletal lesions with considerable variability in histological features and clinical behavior [1]. Although lacking metastatic potential, some can exhibit locally aggressive growth, leading to progressive cortical thinning, bone destruction, deformity, and even pathological fractures, ultimately compromising local function [1,2]. These lesions are more frequent in children, adolescents, and young adults, though they can occur at any age [3]. Clinically, patients may present with nonspecific pain or swelling, whereas in some instances, the tumor may be detected incidentally on imaging. Advanced presentations include deformity or pathological fractures, causing severe pain and functional impairment [1]. Conventional radiographs, MRI, and CT are essential for characterizing morphology, extent, and aggressiveness, guiding the diagnostic pathway [1,4], while biopsy remains the gold standard for definitive diagnosis [5].

A wait-and-see approach can be considered for small, non-aggressive lesions, but surgery is generally indicated for large, fast-growing, or diagnostically uncertain tumors, or when fracture risk is high [6,7]. Intralesional curettage is a well-established surgical technique for most large benign bone tumors [6,7]. The procedure removes the lesion while preserving healthy bone [8,9]. However, curettage alone carries substantial recurrence rates, reaching up to 50% in locally aggressive lesions such as giant cell tumor of bone (GCTB) [10,11]. Various adjuvants have therefore been introduced to eradicate residual tumor cells and reduce recurrence, including high-speed burring, phenol, hydrogen peroxide, alcohol, polymethylmethacrylate (PMMA) cement, and thermal ablation techniques [12,13,14].

Among these, intraoperative cryotherapy, or cryosurgery, has emerged as one of the most promising local adjuvants in managing locally aggressive bone tumors [15,16]. The method employs liquid nitrogen delivered by pour, spray, or closed systems [17]. Controlled freeze–thaw cycles induce tumor necrosis through intracellular ice formation, osmotic damage, and ischemic effects from vascular stasis. By penetrating cancellous bone beyond the reach of mechanical curettage, cryotherapy targets microscopic residual cells, potentially reducing recurrence while maintaining structural integrity [18,19,20].

Despite decades of sporadic clinical use and multiple single-center reports, evidence regarding cryotherapy’s efficacy and safety remains fragmented [17,21,22,23,24,25,26,27,28,29,30,31,32,33,34,35,36,37,38,39,40,41]. Furthermore, concerns persist about potential cryo-induced complications such as fractures, delayed healing, skin necrosis, and neurovascular injury, described in both percutaneous and open procedures [42].

Although several literature reviews have already summarized outcomes with different intraoperative local adjuvants after curettage, to our knowledge, none have focused solely on cryotherapy. This systematic review aims to fill a gap in the literature by evaluating the effectiveness of curettage and cryotherapy in treating benign and low-grade malignant bone tumors.

Our research focuses on investigating the oncological efficacy of the treatment in terms of local recurrences, assessing the safety of the procedure, evaluating complication rates, and evaluating functional outcomes in terms of pain control, functional scores, and motion performance after surgery. This effort aims to orient surgical decision-making and further define the role of cryotherapy in contemporary orthopedic oncological practice.

## 2. Materials and Methods

A systematic review of the literature was performed according to the Preferred Reporting Items for Systematic Reviews and Meta-Analyses (PRISMA) guidelines, using a PRISMA checklist and algorithm [43]. 

### 2.1. Search Strategy, Inclusion and Exclusion Criteria

A comprehensive search was conducted across the PubMed and MEDLINE databases using various combinations of the keywords “cryotherapy,” “cryosurgery”, “bone tumor”, and “sarcoma” [Search string: ((Cryotherapy) OR (Cryosurgery)) AND ((Bone Cyst) OR (Bone Tumor) OR (Sarcoma))]. We included papers published between January 2000 and January 2025. The research was conduced in August 2025. Two independent reviewers (E.I., A.D.A.) conducted the research separately, screening for all the original articles reporting on the surgical treatment of benign and low-grade malignant bone tumors that have been treated with intralesional curettage and cryotherapy as an adjuvant treatment during surgery. The investigators separately reviewed each publication. All articles were initially screened for relevance by title and abstract, excluding articles without an abstract. All potentially suitable articles were then obtained and closely read, with data extracted to minimize selection bias and errors. Only original articles from peer-reviewed journals were included. Inclusion criteria were (1) a confirmed diagnosis of benign, or low-grade malignant bone tumor, (2) curettage, and detailed use of cryosurgery as an adjuvant treatment, along with (3) details on the clinical and oncological outcomes of the received treatments. Pre-clinical studies, literature reviews, articles that did not mention or provide data on cryosurgical treatment, and papers written in languages other than English were excluded. Considering the limited number of articles and the low level of evidence in the few available articles, we included in our study articles ranging from Level I to Level IV. Reports on single cases were excluded. 

**Figure 1:**
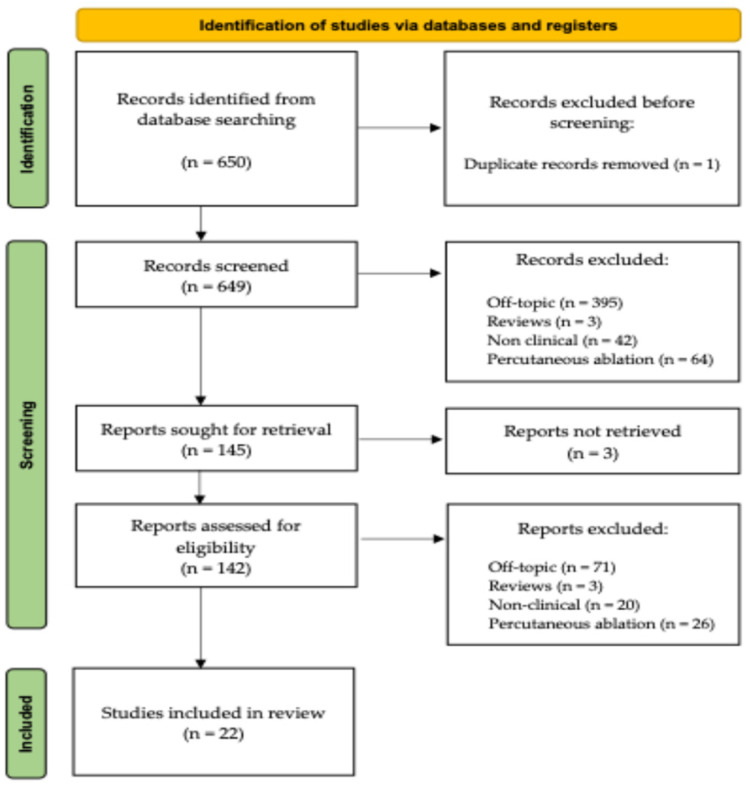
A schematic representation of our study’s PRISMA flow-chart.

The systematic review has been registered online using the INPLASY portal (registration number INPLASY2025100099). The search flowchart, as outlined in the PRISMA guidelines [43], is reported in Figure 1. 

### 2.2. Data Collection for Review Purpose

The year of publication was recorded for each article that met our inclusion criteria. The number of cases included in each study was reported, along with the histological diagnosis of each treated lesion. The mean lesion size (larger diameter) was mentioned when reported. The curettage technique and the eventual use of a high-speed burr or chemical adjuvants other than cryotherapy to clear the surfaces of tumor residuals were described. The technique of choice for cryotherapy administration was considered and recorded. The follow-up of all included cases was considered, and their mean duration was reported for each study. The occurrence and the diagnostic timing of recurrences were considered for each article when available. Functional outcomes, when reported according to the Musculoskeletal Tumor Society (MSTS) scoring system, were also recorded. Intra-operative or post-operative complications, when documented by the authors of the single articles, were recorded in number and type. The absence of information regarding complications, MSTS score, or data on patients’ clinical conditions at their latest follow-up did not constitute exclusion criteria. However, mean and average values were calculated excluding articles that were missing such data. 

### 2.3. Quality Assessment

To account for the heterogeneity in study design and methodology among the selected studies, the Joanna Briggs Institute (JBI) Critical Appraisal tools were employed to critically assess their quality for inclusion in this systematic review. Each item on the checklist is rated with one of four possible responses: “yes”, “no”, “unclear”, or “not applicable” [44].

### 2.4. Statistical Analysis

Statistical analyses were carried out using Stata SE 13.1 (StataCorp LLC, College Station, TX, USA). The complication and local recurrence rates of each study were noted or calculated. The studies’ heterogeneity was calculated, and forest plots were designed for both complications and local recurrences. The size, heterogeneity, and retrospective nature of the included studies discouraged us from setting our review as a meta-analysis. The rarity of bone tumors and the relative paucity of studies on intraoperative cryotherapy led to the inclusion of diverse study types, resulting in heterogeneous data on histological diagnosis, tumor location and size, and surgical techniques. Furthermore, the focus of each study may vary, potentially affecting the reported outcomes.

However, the authors conducted a random-effects analysis to examine complications and local recurrences across the researched studies. Egger tests were used to determine the risk of publication bias for complications and recurrences. Generative artificial intelligence (GenAI) (ChatGPT 5.0, OpenAI Inc., San Francisco, CA, USA) has been used to generate graphics based on our collected data and to double-check statistical analysis.

## 3. Results

A total of 650 articles were identified from the database research. Except for one case of duplication, the titles and abstracts of the remaining 649 articles were evaluated for a first screening. After the screening, 142 available full-text articles were reviewed by both reviewers. Seventy-one of them were off topic (not involving the musculoskeletal apparatus or the use of intra-operative cryotherapy), 20 were not clinical articles (not focusing on the surgical use or the direct prognostic impact of cryosurgery and associated treatments on living patients), and 26 reported on the use of percutaneous cryoablation rather than cryotherapy as an adjuvant treatment in an open surgery scenario. Three additional articles were excluded because they were review articles without documentation of new, original cases.

The remaining 22 articles were included in our review for data collection and statistical investigation [17,21,22,23,24,25,26,27,28,29,30,31,32,33,34,35,36,37,38,39,40,41].

All articles were retrospective, being case series cohort studies.

A summary of the data from all included articles is presented in Table 1.

### 3.1. Quality Assessment

Being retrospective, all the included articles were potentially subject to selection, publication, and reporting biases. The quality of all included papers was assessed against JBI standards. Nine of the 22 articles had “yes” to all ten queries of the JBI checklist. Only two articles were found to be unclear in at least one of the areas investigated by the checklist, but were still considered worthy of being maintained in our review. The remaining 11 articles were found to satisfy all queries completely, except for the last one. However, the lack of advanced statistical analysis in these studies was attributed to the small size of their cohorts.

The JBI quality assessment of all the included articles was reported in detail in Table 2.

### 3.2. Cases

**Figure 2:**
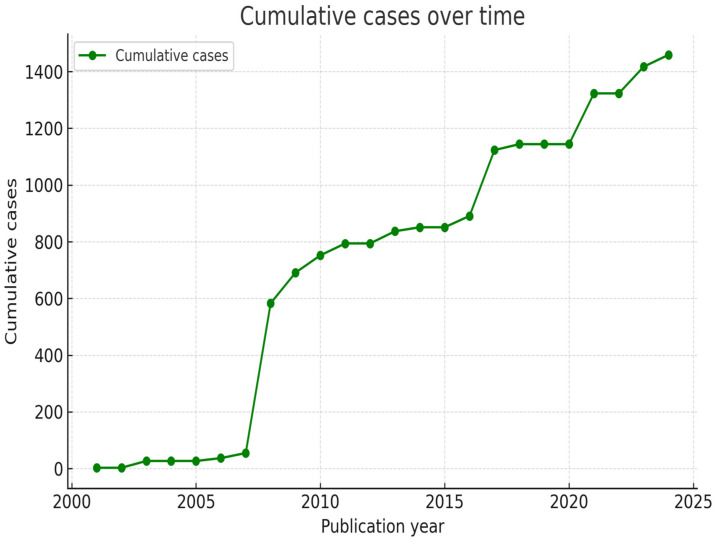
Graphic representation of the cumulative number of reported cases (green line) between January 2000 and August 2025.

A total of 1451 cases were described within the 22 articles that met our inclusion criteria [17,21,22,23,24,25,26,27,28,29,30,31,32,33,34,35,36,37,38,39,40,41]. The mean number of patients per article was 66.3 (3–405). The distribution of cases through our investigation time span is pictured in Figure 2.

### 3.3. Diagnoses

**Figure 3:**
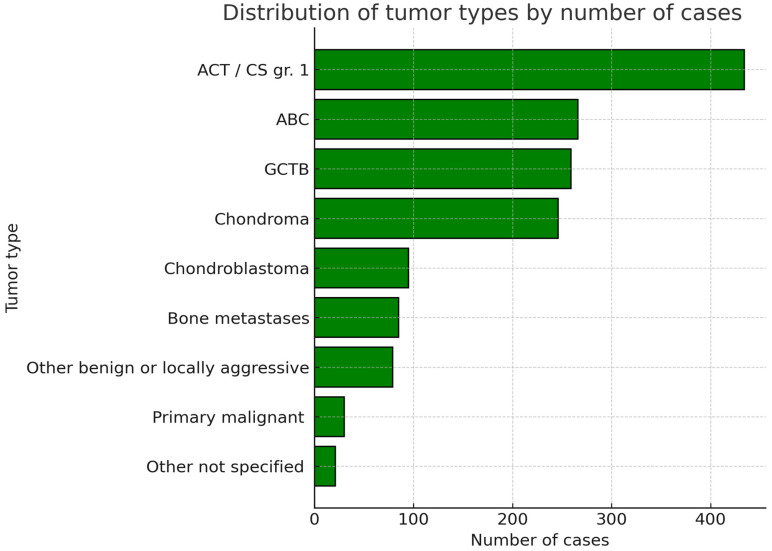
Graphic representation of the cumulative number of included patients sorted per tumor type. ACT/CS gr.1 = Atypical chondromatous tumor/Chondrosarcoma grade 1. ABC = Aneurysmal Bone Cyst. GCTB = Giant Cell Tumor of the Bone.

All the authors of the included articles provided information on the histologic diagnosis of treated patients [17,21,22,23,24,25,26,27,28,29,30,31,32,33,34,35,36,37,38,39,40,41]. A total of 526 patients were diagnosed with either giant cell tumor of the bone (GCTB) or aneurysmal bone cyst (ABC). In particular, 164 cases were diagnosed with GCTB, 171 with ABC, and an additional 190 cases were either diagnosed with one or the other, without further distinction. Ninety patients were diagnosed with chondroblastoma. A total of 680 cases suffered from either chondroma or grade 1 chondrosarcoma/atypical chondromatous tumor (ACT). Two hundred nine cases had chondromas, 396 had ACT, and 75 had either one or another without further distinction. The other tumor types were less common in the reviewed articles. A sum of 79 other benign and low-grade malignant bone tumors was reported. Thirty cases had primary malignant bone tumors. Eighty-five cases with metastatic bone lesions were also included. Finally, the exact histological diagnosis was not specified in 21 cases [24] (Figure 3).

### 3.4. Surgical Treatment and Cryotherapy

**Figure 4:**
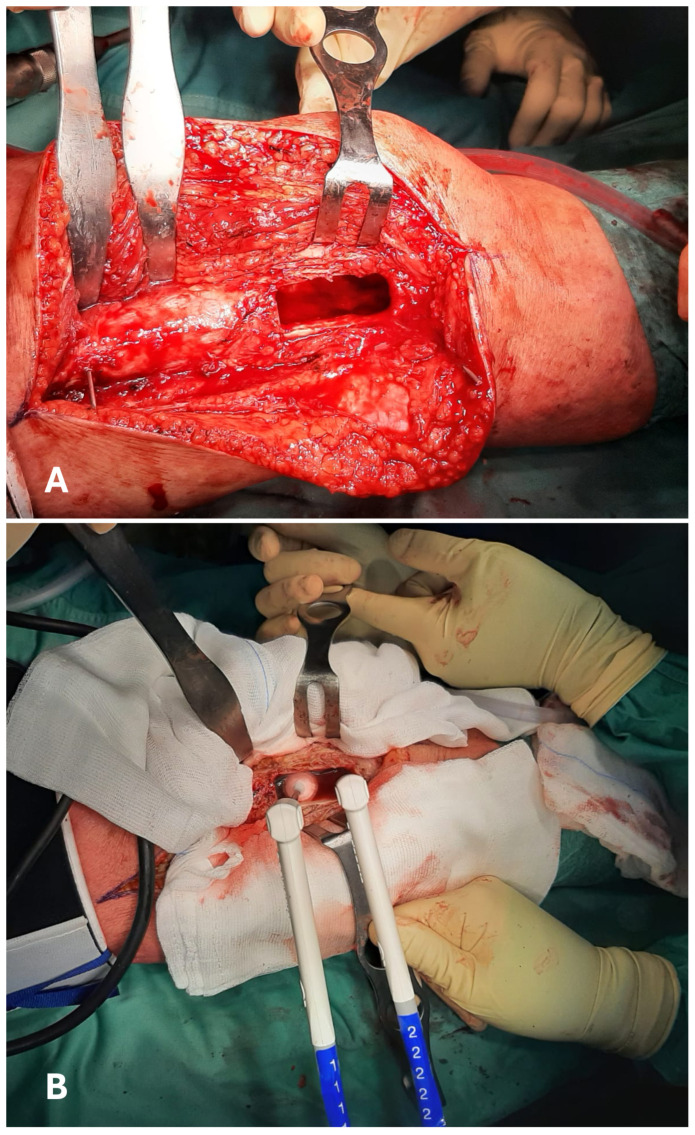
Intraoperative images of a curettage in the distal shaft of a femur (**A**). The surfaces of the resulting cavity are then treated with cryotherapy, administered using two closed-circuit cryoprobes. An ice-ball can be displayed at the tip of the left probe (**B**).

All the authors provided information on the surgical treatment received by their patients. All cases received intralesional curettage [17,21,22,23,24,25,26,27,28,29,30,31,32,33,34,35,36,37,38,39,40,41]. Cryotherapy was always performed after the curettage phase. A total of 1284 cryosurgical treatments were described in detail. The administration strategy relied on the direct pouring of liquid nitrogen in 582 cases [17,21,24,26,31,32,33,37,40,41]. In 552 cases, liquid nitrogen for cryotherapy was administered in spray form [17,26,27,29,35,38,39]. One hundred fifty cases were treated with closed-circuit cryoprobes [22,23,24,25,30,32,34,36] (Figure 4).

**Figure 5:**
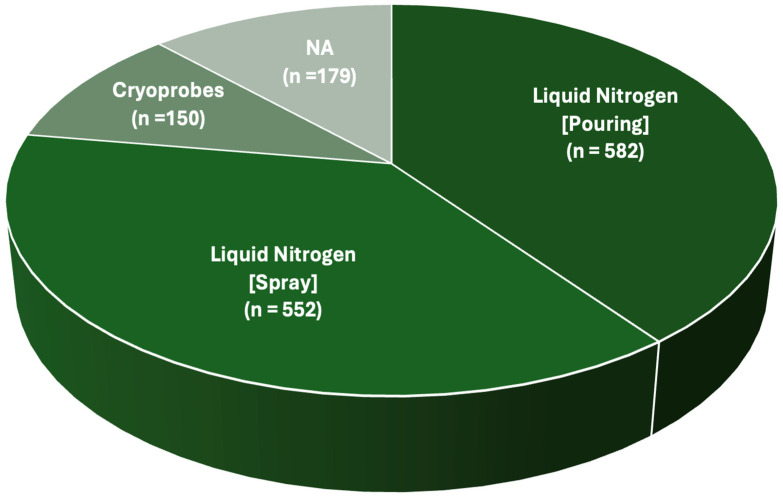
Pie chart describing the total number of patients treated with a certain cryosurgical technique. NA = No data available.

Finally, the cryosurgical technique was not specified for 179 cases [28]. A graphical representation of the global number of patients treated with cryotherapy, sorted per surgical technique, is available in Figure 5.

### 3.5. Post-Operative Follow-Up

Twenty-one of twenty-two articles provided data regarding the mean duration of their patients’ follow-up [17,21,22,23,25,26,27,28,29,30,31,32,33,34,35,36,37,38,39,40,41]. The weighted average post-operative follow-up of the 1227 patients whose timing was available was 55.7 months (12–122).

### 3.6. Complications

**Figure 6:**
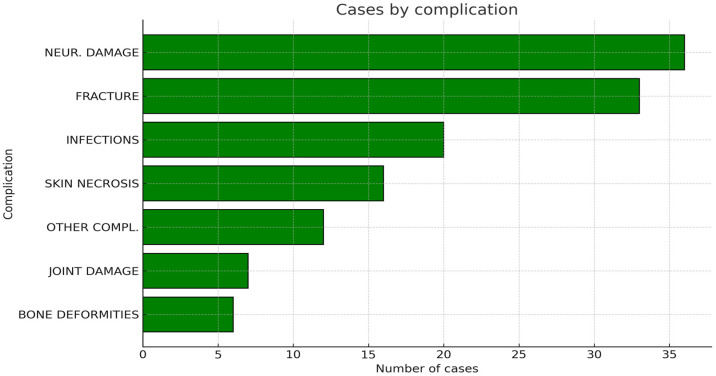
Graphic representation of the cumulative number of complications recorded among all the included articles.

All studied articles described their patients’ post-operative history, either describing or excluding complications [17,21,22,23,24,25,26,27,28,29,30,31,32,33,34,35,36,37,38,39,40,41]. Five articles did not have any complication [22,25,26,37,40], whereas the remaining articles had at least one complicated case [17,21,23,27,28,29,30,31,32,33,34,35,36,38,39,41]. A total of 130 complications were diagnosed after surgery, for an overall complication rate among the evaluated studies of 8.9% (0–33). None of the reported cryosurgical approaches was found to be correlated with a significantly higher complication rate, as determined by a chi-square test (*p* > 0.05). The most common complications were neurological damage (36 cases; 2.5%), fractures (33 cases; 2.3%), infections (20 cases; 1.4%), skin necrosis (16 cases; 1.1%), joint damage (7 cases; 0.5%), and bone deformity (6 cases; 0.4%). Twelve more cases (0.8%) experienced other, less frequent complications (Figure 6).

The remaining 1321 patients were reported to be free of complications at their latest follow-up.

Our data suggested a high degree of heterogeneity in complication rates among the evaluated studies (I^2^ = 70%). According to an Egger test, the risk of publication bias was significantly high (*p* < 0.0001). Forest plots and Funnel plots regarding complications are available as Supplementary Files, as a meta-analysis fell beyond the aims of our review.

### 3.7. Local Recurrences

Data on patients’ oncological outcomes after all the included articles provided surgical treatment [17,21,22,23,24,25,26,27,28,29,30,31,32,33,34,35,36,37,38,39,40,41]. A total of 108 cases suffered from local recurrence after surgical treatment.

The global recurrence rate was 7.4% (0–29). Our data did not reveal statistically significant differences in local recurrence rates between different cryosurgical treatments or between the various primary tumor types (*p* > 0.05). In particular, Meftah et al. [32], who treated a portion of their patients directly by applying liquid nitrogen and another portion by administering it as a spray, reported the same recurrence rate among their treated low-grade chondrosarcomas (9%). No other article allowed a direct comparison. Scoccianti et al. [36] had a significantly higher recurrence rate among the GCBT that received pre-operative treatment with denosumab (29%), compared to those that did not receive such neoadjuvant treatment (11%).

Our data suggested a moderate degree of heterogeneity in complication rates among the evaluated studies (I2 = 28%). According to an Egger test, the risk of publication bias was significantly high (*p* < 0.0001). Forest plots and Funnel plots regarding local recurrences are available as Supplementary Files, as a meta-analysis fell beyond the aims of our review.

### 3.8. Functionality

The functional outcomes of surgical treatment were assessed according to the MSTS scoring system for upper and lower limbs in a total of 463 cases in 11 publications [21,22,23,30,32,33,34,35,37,38,39]. The mean MSTS scores of the single articles ranged between 21.7 and 29. 6. The mean MSTS score of the combined populations was 27.8 (10–30).

## 4. Discussion

Intralesional curettage represents the treatment of choice for many benign and low-grade malignant bone tumors, although its relatively high recurrence rate has led orthopedic oncologists to develop and employ various local adjuvants to eradicate microscopic residuals of disease. [13,45,46,47]. The introduction of high-speed burrs allowed surgeons to overcome sclerotic bone areas and access irregular tumor margins, but excessive use may compromise bone stock and structural stability [6,48]. Chemically active agents such as phenol and hydrogen peroxide have also been used for local tumor control [29,49,50]. Despite their cytotoxic properties, these substances can damage healthy tissues if leakage occurs, and the absence of large long-term studies limits confirmation of their efficacy [51].

Cryotherapy was first introduced in orthopedic oncology by Marcove and Miller [52,53] in the late 60 s to induce tissue necrosis while preserving mineralized bone. Cryosurgery is now one of the most used local adjuvants to curettage [13,17,21,22,23,24,25,26,27,28,29,30,31,32,33,34,35,36,37,38,39,40,41]. The treatment induces necrosis through rapid freezing followed by gradual thawing. Ice crystal formation damages cell membranes and cytoplasmic structures, while subsequent microvascular injury triggers coagulative necrosis. Repeated freeze–thaw cycles amplify these effects, maximizing cryo-induced tissue destruction [52,53,54,55,56]. 

Different cryosurgical techniques have been developed. The most commonly used include direct application of liquid nitrogen, its administration as a spray, and the use of computerized closed cooling systems [13,17,21,22,23,24,25,26,27,28,29,30,31,32,33,34,35,36,37,38,39,40,41]. For decades, direct pouring was standard practice, proving effective but requiring precise handling to prevent nitrogen leakage and collateral damage to surrounding tissues [17,21,24,26,31,32,33,37,40,41]. To improve safety and precision, protective measures such as wet gauzes and improved applicators were introduced [17]. The spray technique, delivered through maneuverable or rigid tubes, reduces direct exposure to liquid nitrogen while achieving effective cooling, and has become a reliable, well-established option [17,26,27,29,35,38,39]. More recently, closed-system cryotherapy has gained popularity in surgical oncology. These computerized systems utilize cryoprobes that generate ice balls under controlled conditions, eliminating the need to handle liquid nitrogen directly [22,23,24,25,30,32,34,36]. Though expensive, this technology offers excellent precision and control of local temperatures and freeze–thaw cycles [25,57], potentially integrating with future advances such as intraoperative navigation and augmented reality.

Our review indicates that the combination of curettage and cryotherapy, performed using any of the three techniques, results in reasonably low recurrence rates. The global recurrence rate of almost 1500 cases, mainly diagnosed with benign and low-grade malignant tumors, was as low as 7.4% (0–29) [13,17,21,22,23,24,25,26,27,28,29,30,31,32,33,34,35,36,37,38,39,40,41]. All but one study reported recurrence rates lower than 12% [13,17,21,22,23,24,25,26,27,28,29,30,31,32,33,34,35,37,38,39,40,41]. The remaining paper, published by Scoccianti et al. [36], had a recurrence rate of 29% for the GCBTs that received pre-operative treatment with denosumab (29%). In contrast, only 11% of those who received only curettage and cryotherapy developed local recurrences. Altogether, our data suggest that cryotherapy is an effective local adjuvant treatment for bone tumors after intralesional curettage.

For a long time, intra-operative and post-operative complications have been considered some of the biggest concerns for the widespread adoption of cryotherapy in orthopedic oncology. Well-known complications in cryosurgery include skin necrosis, wound dehiscence, and neurological damage, but also long-term bone weakening with pathological fractures, deformities, and even joint degeneration. Although many of these adverse conditions had been documented in literature over the last quarter century, our review suggests a cumulative complication rate as low as 8.9% [13,17,21,22,23,24,25,26,27,28,29,30,31,32,33,34,35,36,37,38,39,40,41]. In particular, neurological damage (rate of 2.5%) and post-operative fractures (rate of 2.3%) emerged as the most frequent complications. The risk of neurological deficits could be further reduced by avoiding direct contact between the cooling source and the nerve and its nearby structures, covering the soft tissues, and eventually irrigating them with mild hot water [17]. Plate stabilization after adequate filling of the bone cavity may also minimize the risk of postoperative fractures following curettage and cryotherapy [58]. All in all, the reasonable complication rates found in our review support the reliability and safety of cryosurgery for the open treatment of musculoskeletal tumors.

Furthermore, another indication of the effectiveness of curettage and intraoperative cryotherapy is the favorable performance outcomes reported in several articles included in our review. The mean MSTS score of 463 cases from 11 studies was 27.8/30 (10–30) [21,22,23,30,32,33,34,35,37,38,39]. Such good post-operative functionality should be considered a primary outcome, especially in young patients with bone tumors, whose quality of life should not be underestimated in a mid to long-term scenario [59,60].

The outcomes of our review suggest that cryotherapy should be considered as a local adjuvant, especially in lesions with complex shape, large size, or proven local aggressiveness [2,6,7,8,9]. Pre-operative planning can be performed using common radiological images or sophisticated technologies such as augmented reality [25]. After a vigorous and accurate curettage performed with spoons alone or with high-speed burrs, cryotherapy could be administered using any of the three techniques described in our paper. Before treatment begins, soft tissues—particularly skin, vessels, and nerves—should be carefully covered to avoid direct contact with the freezing source. As noble soft tissues are secured and shielded, cryotherapy can be performed [28,29,30,31,32,33,34,35,36,37,38,39,40,41]. All articles agree that at least two freeze-thaw cycles are necessary to maximize the treatment’s effectiveness [17,21,22,23,24,25,26,27,28,29,30,31,32,33,34,35,36,37,38,39,40,41]. Regardless of the chosen technique, the entire surface left by curettage should be covered. To do so, surgeons could take advantage of gravity and adjust the inclination of the treated bone during a pouring treatment or change the disposition of the cryoprobes between cycles. Finally, after surgery, radiological and careful clinical evaluations should be performed to confirm the absence of postoperative complications [29,46].

We acknowledge that our study has some limitations. The rarity of bone tumors limited the number of case series and the size of their cohorts, thereby limiting the reliability and significance of available data. To increase the number of patients, some authors gathered cases treated with different diagnoses arising in various locations. The retrospective nature of the included studies represented another limitation. Such a design implied a relatively low level of standardization of care, with patients treated with heterogeneous surgical techniques, potential discrepancies in data collection, and possible recall bias. The absence of prospective studies, along with the high heterogeneity among the included articles, limited the reliability of some of our data. In fact, the inclusion of low-evidence studies, which were the only available at the moment, introduced a potential bias into our review. These limits could be overcome in the near future with large-scale prospective studies on the topic. The absence of control arms impedes the ability to attribute outcomes to cryosurgical treatment with certainty.

Beyond these limitations, our research provides an unprecedented overview of the clinical and oncological outcomes associated with the combination of curettage and intraoperative cryotherapy. The authors who added one or more cycles of freezing and thawing after the curettage had relatively low rates of local recurrence, even for locally aggressive or low-grade malignant bone tumors. These outcomes, characterized by reasonable complication rates and good postoperative functional performance in the treated limbs, support the effectiveness of cryotherapy as a local adjuvant in orthopedic oncology surgery.

## 5. Conclusions

The combination of curettage and intra-operative cryotherapy, administered with either open or closed contact techniques, can be effective in eradicating benign and low-grade malignant bone tumors, with low complication rates and a limited impact on patients’ post-operative functionality.

## Figures and Tables

**Table 1:** A summary of all the main data contained in the articles included in our review.

Authors	Surgical Treatment	N	Tumor Type	Complications (%)	LR (%)	MSTS	FU (m)
Scoccianti et al. [36] (2018)	Curettage + Cryotherapy (Cryoprobes)	21	Giant Cell Tumor of Bone	0	29%	-	39
Chen et al. [24] (2017)	Curettage + Cryotherapy (212 Poured LN, 2 Cryoprobes)	232	Chondroblastoma (16), Chondromyxoid Fibroma (7), Osteoblastoma (1), Nonossifying Fibroma (8), Enchondroma (75), Low-Grade Chondrosarcoma (1), Aneurysmal Bone Cyst (20), Giant Cell Tumor (42), Unicameral Bone Cyst (7), Lipoma (2), Fibrous Dysplasia (3), Eosinophilic Granuloma (1), Other Metastatic (19), Other Primary Malignant Tumors (9), Other (21)21	2% (3 Infections, 1 Fracture, 1 Neurological damage)	6%	-	NA
Yurtbay et al. [41] (2023)	Curettage + Cryotherapy (Poured LN)	30	Chondroblastoma	23% (2 Joint degeneration, 2 Physeal growth arrest, 3 Skin necrosis)	7%	-	54
Meller et al. [17] (2008)	Curettage + Cryotherapy (200 Poured LN, 240 Spray LN)	405	Giant Cell Bone Tumor/Aneurysmal Bone Cyst (190), Chondroblastoma (20), Chondromyxoid fibroma (6), Osteoblastoma (2), Hemangioma (1), Schwannoma (1), Fibrous dysplasia (20), Ossifying fibroma (1), Desmoblastic fibroma (1), Eosinophilic granuloma (4), Enchondroma and Low-Grade Chondrosarcoma (75), Hemangioendothelioma (2), Chordoma (2), Ependymoma (1), Ewing’s sarcoma (9), Osteosarcoma (7), Myeloma (5), Renal cell carcinoma 21, Other metastasesc (37)	5% (9 Infection, 4 Fractures, 6 Skin necrosis, 3 Neurological deficit)	8%	-	84
Deckers et al. [28] (2021)	Curettage + Cryotherapy (NA)	179	Enchondromas (55), Low-Grade Chondrosarcoma (119), Chondrosarcoma Grade 2 (5)	13% (Neurological deficit 23, Infection 1)	12%	-	54
Park et al. [34] (2023)	Curettage + Cryotherapy (Cryorobes)	44	Low-Grade Chondrosarcoma	16% (4 Implant failure, 2 Articular degeneration, 1 Fracture)	9%	21.7 / 27	94
Levanon et al. [30] (2024)	Curettage + Cryotherapy (Cryorobes)	42	Aneurysmal Bone Cyst	17% (4 Deformities, 3 Not mentioned)	7%	27.8	73
Mohler et al. [33] (2010)	Curettage + Cryotherapy (Poured LN)	46	Low-Grade Chondrosarcoma	6% (3 Fractures)	4%	27.2	40
Peeters et al. [35] (2009)	Curettage + Cryotherapy (Spray LN)	80	Aneurysmal Bone Cyst	6% (3 Neurological deficit, 1 Fracture, 1 Infection)	5%	29.2	55
Meftah et al. [32] (2013)	Curettage + Cryotherapy (32 Poured LN, 11 Cryoprobes)	43	Low-Grade Chondrosarcoma	7% (2 Fractures, 1 Infection)	9%	26.7	122
Mashhour et al. [31] (2014)	Curettage + Cryotherapy (Poured LN)	14	Chondroblastoma	21% (2 Joint degeneration, 1 Skin necrosis)	7%	-	49
van der Geest et al. [39] (2008)	Curettage + Cryotherapy (Spray LN)	123	Chondroma (75), Low-Grade Chondrosarcoma (55)	20% (18 Fracture, 3 infection, 3 Neurological deficit, 1 Gas embolyzation)	2%	28	60
Wittig et al. [40] (2001)	Curettage + Cryotherapy (Poured LN)	3	Giant Cell Tumor of Bone	33% (1 Skin necrosis)	0	-	54
Ahlmann et al. [22] (2006)	Curettage + Cryotherapy (Cryoprobes)	10	Giant Cell Tumor of Bone	10% (1 Fistula)	0	27.3	38
Souna et al. [37] (2010)	Curettage + Cryotherapy (Poured LN)	15	Low-Grade Chondrosarcoma	0%	0	27.9	96
van der Geest et al. [38] (2007)	Curettage + Cryotherapy (10) (Spray LN) Curettage alone or Resection (8)	10	Chondroblastoma	20% (1 Articular damage, 1 Blisters from nitrogen leakage)	10%	29	60
Dabak et al. [26] (2003)	Curettage + Cryotherapy (7 Poured LN, 17 Spray LN)	24	Aneurysmal Bone Cyst (11), Giant cell Tumor of Bone (6), Chondroma (4), Metastases (3)	17% (3 Skin necrosis, 1 Infection)	0	-	47
Lee et al. [29] (2011)	Curettage + Cryotherapy (Spray LN)	42	Low-Grade Chondrosarcoma (29), Giant Cell Tumor of Bone (8), Aneurysmal Bone Cyst (4), Hemangioendothelioma (1)	10% (3 Neurological deficit. 1 Heterotopic ossification)	12%	-	22
Andreani et al. [23] (2023)	Curettage + Cryotherapy (Cryoprobes)	14	Aneurysmal Bone Cyst	7% (1 Wound Dehiscence)	7%	29.6	38
Dabak et al. [27] (2016)	Curettage + Cryotherapy (Spray LN)	40	Giant Cell Tumor of Bone	5% (1 Cement loosening, 1 Fracture)	7%	-	43
Abdelrahman et al. [21] (2009)	Curettage + Cryotherapy (Poured LN)	28	Giant Cell Tumor of Bone	14% (1 Skin necrosis, 2 Fracture, 1 Infection)	4%	28.2	34
D’Arienzo et al. [25] (2023)	Curettage + Cryotherapy (Cryoprobes)	6	Giant Cell Tumor of Bone	0	0	-	12

N = Number. FU = Follow-Up. (m) = Month. NA = Not available.

**Table 2:** The answers to all the queries of the JBI checklist for case series studies.

Authors	Q1	Q2	Q3	Q4	Q5	Q6	Q7	Q8	Q9	Q10
Scoccianti et al. (2018) [36]	Yes	Yes	Yes	Yes	Yes	Yes	Yes	Yes	Yes	Yes
Chen et al. (2017) [24]	Yes	Yes	Yes	NA	Yes	Yes	Yes	Unclear	Yes	NA
Yurtbay et al. (2023) [41]	Yes	Yes	Yes	Yes	Yes	Yes	Yes	Yes	Yes	Yes
Meller et al. (2008) [17]	Yes	Yes	Yes	Yes	Yes	Yes	Yes	Yes	Yes	NA
Deckers et al. (2021) [28]	Yes	Yes	Yes	Yes	Yes	Yes	Yes	Yes	Yes	Yes
Park et al. (2023) [34]	Yes	Yes	Yes	Yes	Yes	Yes	Yes	Yes	Yes	NA
Levanon et al. (2024) [30]	Yes	Yes	Yes	Yes	Yes	Yes	Yes	Yes	Yes	Yes
Mohler et al. (2010) [33]	Yes	Yes	Yes	Yes	Yes	Yes	Yes	Yes	Yes	NA
Peeters et al. (2009) [35]	Yes	Yes	Yes	Yes	Yes	Yes	Yes	Yes	Yes	NA
Meftah et al. (2013) [32]	Yes	Yes	Yes	Yes	Yes	Yes	Yes	Yes	Yes	Yes
Mashhour et al. (2014) [31]	Yes	Yes	Yes	Yes	Yes	Yes	Yes	Yes	Yes	NA
van der Geest et al. (2008) [39]	Yes	Yes	Yes	Yes	Yes	Yes	Yes	Yes	Yes	Yes
Wittig et al. (2001) [40]	Yes	Yes	Yes	Yes	Yes	Unclear	Yes	Unclear	Yes	NA
Ahlmann et al. (2006) [22]	Yes	Yes	Yes	Yes	Yes	Yes	Yes	Yes	Yes	NA
Souna et al. (2010) [37]	Yes	Yes	Yes	Yes	Yes	Yes	Yes	Yes	Yes	NA
van der Geest et al. (2007) [38]	Yes	Yes	Yes	Yes	Yes	Yes	Yes	Yes	Yes	NA
Dabak et al. (2003) [26]	Yes	Yes	Yes	Yes	Yes	Yes	Yes	Yes	Yes	NA
Lee et al. (2011) [29]	Yes	Yes	Yes	Yes	Yes	Yes	Yes	Yes	Yes	Yes
Andreani et al. (2023) [23]	Yes	Yes	Yes	Yes	Yes	Yes	Yes	Yes	Yes	Yes
Dabak et al. (2016) [27]	Yes	Yes	Yes	Yes	Yes	Yes	Yes	Yes	Yes	Yes
Abdelrahman et al. (2009) [21]	Yes	Yes	Yes	Yes	Yes	Yes	Yes	Yes	Yes	NA
D’Arienzo et al. (2023) [25]	Yes	Yes	Yes	Yes	Yes	Yes	Yes	Yes	Yes	NA

NA = Not Applicable.

## Data Availability

The data that support the findings of this study are available from the corresponding author upon reasonable request.

## Supplementary Materials

The following supporting information can be downloaded at https://www.mdpi.com/article/10.3390/jcm14228007/s1.
