# Supplementary figures and images for: Intraoperative Cryotherapy as a Local Adjuvant After Bone Curettage in Orthopedic Oncology: A Review of Modern Literature
Source: J Clin Med. 2025 Nov 12;14(22):8007. doi: 10.3390/jcm14228007 (PMC12653735; doi:10.3390/jcm14228007)

# Complications (meta-analysis of proportions, RE-DSL)

$I^2=70.3\%$   $\tau^2=0.0391$

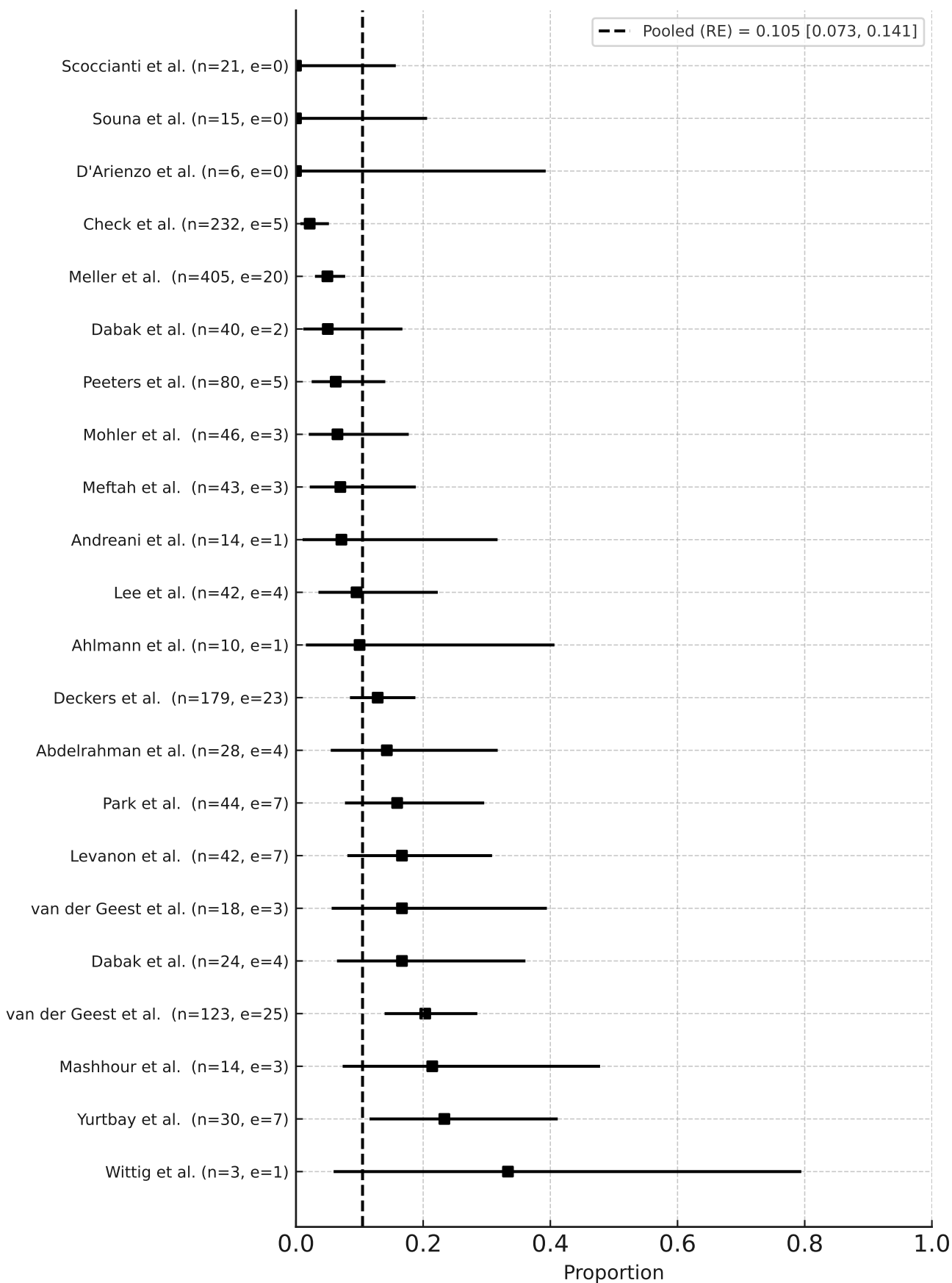

Supplement: Supplementary file 1 [file jcm-14-08007-s001.zip › Forest plot complications.pdf]

Funnel plot - Complicanze

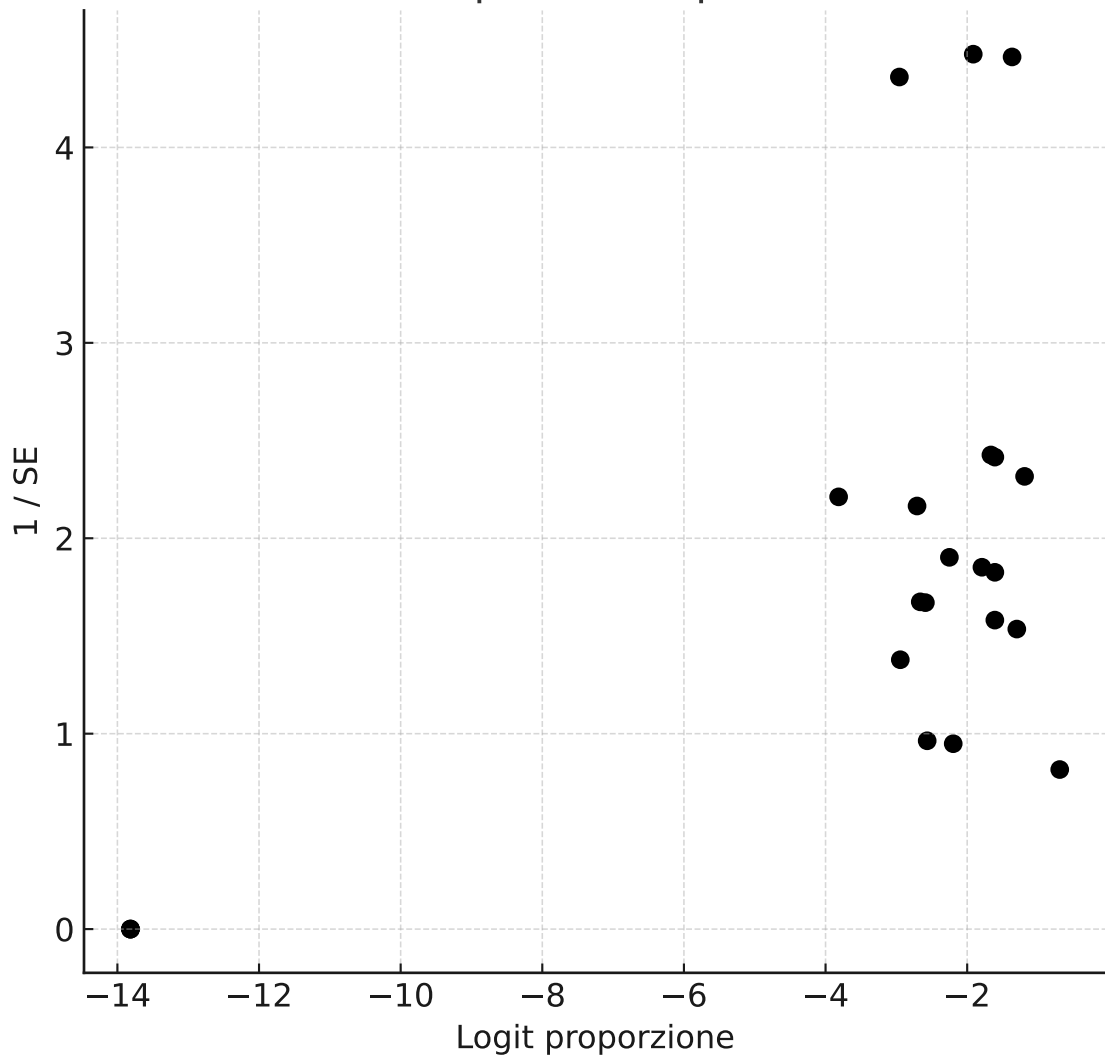

Supplement: Supplementary file 1 [file jcm-14-08007-s001.zip › Funnel plot complication.pdf]

Funnel plot - Recidive

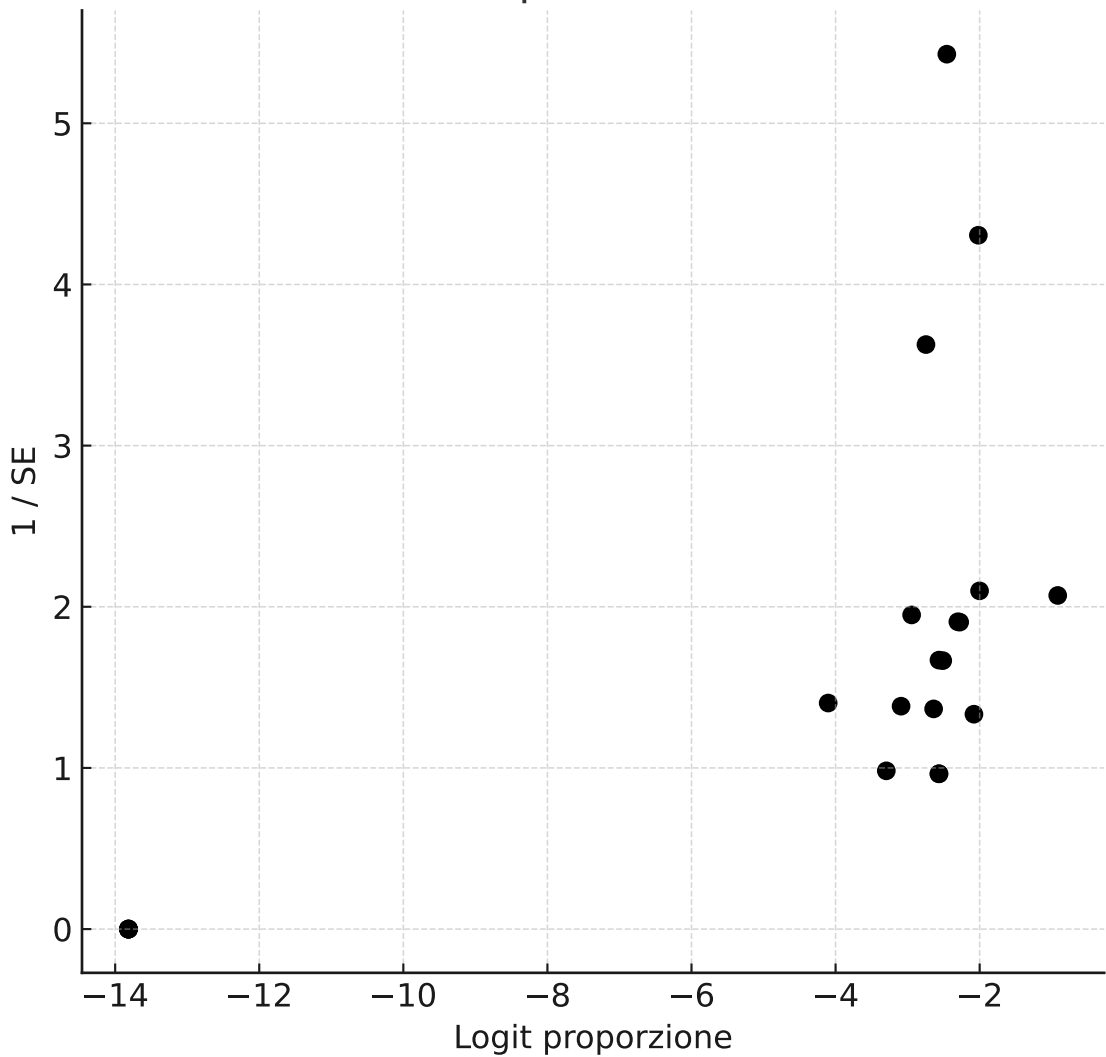

Supplement: Supplementary file 1 [file jcm-14-08007-s001.zip › Funnel plot recurrence.pdf]
